# Mapping Genetic Associations With Functional Brain Area Alterations in Schizophrenia and Implications for Cortical Development

**DOI:** 10.1002/brb3.70688

**Published:** 2025-07-20

**Authors:** Jun‐Ding Zhu, Chih‐Yun Chung, Shu‐Fei Lin, Shih‐Jen Tsai, Albert C. Yang, Pei‐Shan Hou

**Affiliations:** ^1^ Department of Occupational Therapy, College of Medical Science and Technology Chung Shan Medical University Taichung Taiwan; ^2^ Occupational Therapy Room Chung Shan Medical University Hospital Taichung Taiwan; ^3^ Institute of Anatomy and Cell Biology National Yang Ming Chiao Tung University Taipei Taiwan; ^4^ Institute of Brain Science National Yang Ming Chiao Tung University Taipei Taiwan; ^5^ Department of Psychiatry Taipei Veterans General Hospital Taipei Taiwan; ^6^ Brain Research Center National Yang Ming Chiao Tung University Taipei Taiwan; ^7^ Digital Medicine and Smart Healthcare Research Center National Yang Ming Chiao Tung University Taipei Taiwan; ^8^ Department of Medical Research Taipei Veterans General Hospital Taipei Taiwan; ^9^ School of Medicine, College of Medicine National Yang Ming Chiao Tung University Taipei Taiwan

**Keywords:** Brodmann's area, cortical development, genome‐wide association study, Schizophrenia, gray matter volume

## Abstract

**Background:**

While prior studies have identified regional reduction in gray matter (GM) volume in schizophrenia, it remains unclear whether these alterations are concentrated in specific brain functional areas and how they relate to genetic factors. This study aimed to identify Brodmann's areas (BAs) with affected GM volume in individuals with schizophrenia, explore associated genetic variants through a genome‐wide association study (GWAS), and investigate the potential roles of these genes during cortical development.

**Methods:**

The study recruited 194 individuals with schizophrenia and 330 healthy controls from the Taiwan Aging Mental Illness cohort. T1‐weighted MRI scans were processed to assess GM volume changes, and the cerebral cortex was parcellated into BAs for detailed analysis. GWAS was conducted to identify schizophrenia‐associated genetic variants, followed by functional mapping, single‐cell RNA sequencing analysis of developing human cortical cells, and in situ hybridization analysis in the developing mouse neocortex.

**Results:**

Significant reductions in GM volume were found in specific BAs, particularly in the ventral frontal cortex, anterior temporal lobe, and cingulate cortex, with BA13, BA23, BA24, BA25, BA27, BA28, BA31, BA34, BA35, and BA38 showing the most pronounced changes. GWAS identified multiple genetic variants associated with these affected BAs. Further, single‐cell RNA sequencing and in situ hybridization analyses revealed dynamic expression patterns of the schizophrenia‐associated genes during cortical development, suggesting their potential roles in the structural abnormalities observed in schizophrenia.

**Conclusions:**

The findings support the hypothesis that specific BAs are more vulnerable to GM volume reduction in schizophrenia, potentially driven by distinct genetic factors.

## Graphical abstract

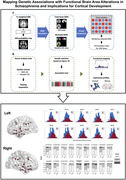

## 1 Background

Schizophrenia is a chronic mental disorder characterized by disturbances in thinking, perception, emotions, and behavior. The prevalence of schizophrenia is approximately 1% worldwide (Holder and Wayhs 2014), and it typically emerges in late adolescence and early adulthood stages. Although the pathogenic mechanism of schizophrenia is not fully understood, accumulating evidence has suggested the polygenetic causes of schizophrenia (Gejman et al. 2010; Lam et al. 2019). Although previous studies have identified various genes potentially associated with schizophrenia, the recurrence rate of these genes is relatively low. This low recurrence rate may contribute to the heterogeneous clinical phenotypes observed in schizophrenia, which are primarily governed by the cerebral cortex. Recent studies have revealed that patients with schizophrenia exhibit uneven alterations in brain gray matter (GM) (Dietsche et al. 2017; Kubota et al. 2011; Lin et al. 2019). However, given that the cerebral cortex comprises multiple functional regions, it remains unclear whether these alterations are enriched in specific functional areas and whether the changes in GM volume within these regions are associated with particular genes.

The cerebral cortex can be parcellated into 52 functional areas, called Brodmann's areas (BA), based on the cytoarchitecture of the GM regardless of the anatomical structures, such as the gyrus (Brodmann 1909). Each BA has a unique cell component responsible for specific biological functions. For example, the BA 4 primary motor area barely has layer IV neurons and sends signals outside the cortex to control voluntary movement (Barbas and García‐Cabezas 2015). Adjacent areas BA 3, 1, and 2 primary somatosensory cortex on the postcentral gyrus have abundant layer IV neurons and receive signals from the thalamus (Miller et al. 2001). To establish the unique neocortical cytoarchitecture, the developmental processes of the neocortex are sophisticatedly regulated. Neocortical neurons were produced in humans during gestational Weeks 7 (GW7) to GW27 (Betizeau and Dehay 2016). During this period, two cues control the corticogenesis. One is the standard mechanism to produce neurons sequentially to form the typical six‐layered laminar structures from superficial Layer I to bottom Layer VI. The other factor is orchestrating the regional‐specific cytoarchitectures used for area parcellation (Brodmann 1909; Toga 2015). Although whether this factor is coming from inside or outside the neocortex is still under debate (Mallamaci and Stoykova 2006; Bhaduri et al. 2021), it controls the cell components in the cerebral cortex to designate biological functions.

MRI was a powerful tool to assist in assessing schizophrenia. The cerebral structures have been found to exhibit abnormalities in structural MRI studies of schizophrenia. In a previous MRI study, the GM volume of individuals with schizophrenia was significantly decreased in the whole brain, especially in the frontal lobe and temporal lobe, and presented enlarged ventricles (Brent et al. 2013; Shenton et al. 2001; Vita et al. 2012; Yue et al. 2016). Nonetheless, the cerebral cortex comprises multiple functional BAs, and the functional areas have unique functions. This fact lets us hypothesize that the changes in GM may be restricted in certain areas, which might result from certain genetic factors. Therefore, in this study, we aimed to (1) identify the BAs with affected GM volume in individuals with schizophrenia compared to healthy controls, (2) explore the schizophrenia‐related BA‐associated genes using a genome‐wide association study and functional mapping, and (3) investigate the possible involvement of schizophrenia‐related BA‐associated genes using scRNA‐sequencing analysis of developing human neocortical cells and RNA expression profiles in the developing mouse neocortex.

## 2 Methods

### 2.1 Participants

A total of 194 individuals with schizophrenia and 330 healthy controls from the “Taiwan Aging Mental Illness (TAMI)” cohort were recruited. Each individual with schizophrenia was diagnosed according to the DSM‐IV‐TR criteria by two psychiatrists. Individuals with schizophrenia were excluded with a history of other psychiatric or neurological disorders. Additionally, healthy controls with any psychiatric or neurological disorders were excluded. Cognitive function was assessed in all participants using the Mini‐Mental State Examination (MMSE) (Folstein et al. 1975). In addition, the Positive and Negative Syndrome Scale (PANSS) was used to assess symptom severity in patients with schizophrenia (Kay et al. 1988). There were no significant differences between sex and age of the two groups (individuals with schizophrenia: 43.25 ± 11.93 years, range: 20–70; healthy controls: 43.49 ± 15.47 years, range: 20–84; *p* = 0.85) and sex (*p* = 0.28) (Zhu et al. 2023). This study was conducted in accordance with the Declaration of Helsinki and was approved by the Institutional Review Board of Taipei Veterans General Hospital and National Yang Ming Chiao Tung University. All participants provided written informed consent.

### 2.2 Image Acquisition and Preprocessing

The T1‐weighted MR scans were acquired by a 3T MRI scanner (Siemens Magnetom Tim Trio, Erlangen, Germany). The raw images were processed using Data Processing Assistant for Resting‐State fMRI (DPARSF) in the DPABI toolbox in MATLAB R2020b (Mathworks, Natick, MA, USA) (Yan et al. 2016). The Supporting Information provides additional information on the scanning protocol and preprocessing steps.

### 2.3 Brodmann Map

**FIGURE 1:**
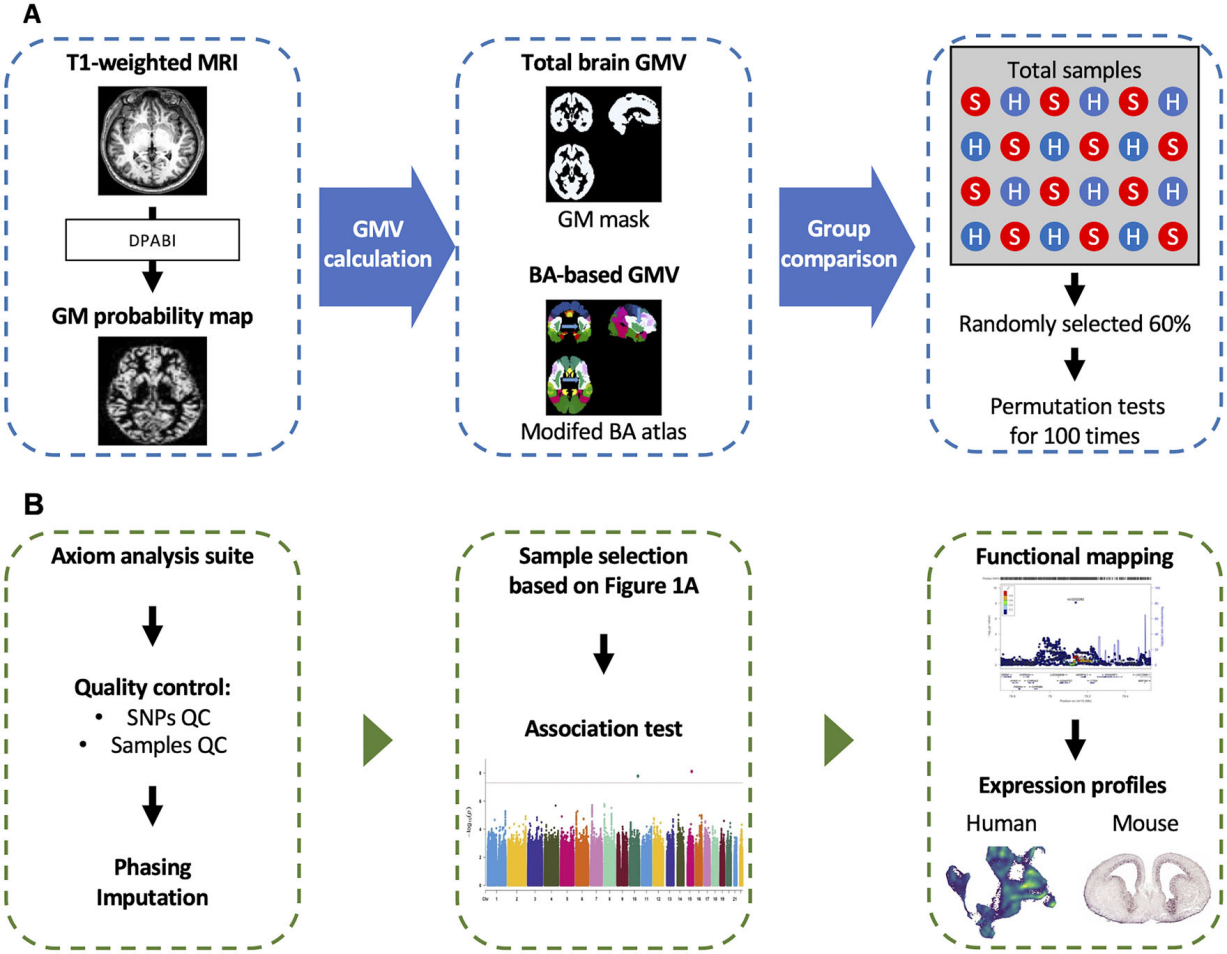
The workflow to identify the affected Brodmann areas and correlated genes in schizophrenia. (A) The workflow of T1‐weighted MRI analysis and permutation tests to identify the affected Brodmann's areas in individuals with schizophrenia. (B) The workflow of genome‐wide association study, functional mapping, and the expression profiles to identify the schizophrenia‐associated genes in cortical development. BA, Brodmann's area; GMV, gray matter volume; H, healthy controls; QC, quality control; S, schizophrenia.

Since the Brodmann interactive area atlas in MRIcro software lacks two areas (https://people.cas.sc.edu/rorden/mricro/mricro.html) (Rorden and Brett 2000) (i.e., the BA13 insula and the BA31 dorsal posterior cingulate cortex), we extracted the information from the Harvard–Oxford cortical and subcortical structural atlases (RRID:SCR_001476) and the automated anatomical labeling atlas (AAL) (Rolls et al. 2020) to generate the modified Brodmann map (Figure 1A). See the Supporting Information for details.

### 2.4 GWAS Genotype Sample Collection and Quality Control

Individual genotypes were examined using the Axiom Genome‐Wide TWB 1.0 Array Plate (Affymetrix) with 653,291 tagged SNPs based on the GRch37/hg19 chromosome. Quality control was performed using PLINK (version 1.9) (Purcell et al. 2007) and PLINK2 (version 2.0) (Chang et al. 2015). See the Supporting Information for details. A total of 404 samples, with 232 controls and 172 cases (183 males and 221 females), and 590,776 SNPs were obtained.

### 2.5 Haplotype Phasing and Genotype Imputation

Haplotype phasing and genotype imputation were performed to improve the statistical power using Eagle (version 2.4.1, https://alkesgroup.broadinstitute.org/Eagle) (Loh, Danecek, et al. 2016; Loh, Palamara, et al. 2016) on the Michigan Imputation Server (version 1.0.2, https://imputationserver.sph.umich.edu) (Das et al. 2016) and Impute5 (Rubinacci et al. 2020), respectively, with The 1000 Genomes Project Consortium (2015) phase 3v5 East Asian population panel as references. A total of 5,813,972 SNPs were obtained.

### 2.6 Genome‐Wide Association Test, GWAS, of Identified Schizophrenia‐Related BA Phenotype

The individuals with schizophrenia with a significant decrease of GM volume in the identified BAs were selected using one‐sample *t*‐tests with all healthy controls as the controls. GWAS tests were performed using logistic regression in PLINK. The SNPs with a *p* value less than 5 × 10^−6^ were identified as the schizophrenia‐associated SNPs. Odds ratio was calculated in PLINK. The GWAS results were visualized using an rMVP R package (Yin et al. 2021).

### 2.7 Functional Annotation of SNPs to Genes

To further investigate the potential function of the schizophrenia‐associated SNPs, the Functional Mapping and Annotation of Genome‐Wide Association Studies server (FUMA GWAS) (Watanabe et al. 2017, 2019) was used to map the associated SNPs into genes.

### 2.8 Polygenic Risk Score

PRS calculations were based on GWAS data from the Psychiatric Genomics Consortium, involving 22,778 schizophrenia cases and 35,362 East Asian controls (Lam et al. 2019). QC steps included filtering variants and removing duplicate and ambiguous SNPs. PRS was calculated using PRSice‐2 (Choi and O'Reilly 2019) with linkage disequilibrium clumping using the 1000 Genomes Project East Asian panel as the reference. The optimal *p* value threshold was identified through regression analysis, and the BA‐based PRS was standardized and visualized using MATLAB's “histfit” function. See the Supporting Information for details.

### 2.9 Single‐Cell RNA Sequencing Analysis

RNA counts matrices from embryonic somatosensory, motor, parietal, temporal, visual, and prefrontal areas at gestation week (GW) 20 were downloaded from the database provided by the Kriegstein laboratory in UCSF (https://data.nemoarchive.org/biccn/grant/u01_devhu/kriegstein/transcriptome/scell/10x_v2/human/processed/counts/) (Bhaduri et al. 2021). The data was analyzed using a Seurat v4.0 R toolkit with RStudio software (https://satijalab.org/seurat/). A total of 35 principal components were used to generate UMAP. A Nebulosa R toolkit was used to visualize the density of RNA expression.

### 2.10 Sample Collection and Cryosection

Institute of Cancer Research (ICR) mice were utilized and housed at the National Yang Ming Chiao Tung University Laboratory Animal Center following the guidelines of the Institutional Animal Care and Use Committee (IACUC). The detection of a vaginal plug marked as embryonic day 0.5, E0.5, and the postnatal day 0, P0, corresponding to the day of birth. Brains were collected from three stages (E14.5, E18.5, and P4). Serial coronal sections were prepared using a Thermo Cryostat. See the Supporting Information for details.

### 2.11 In Situ Hybridization

RNA probes were generated using nested PCR to amplify the sequence of the genes of interest, followed by in vitro transcription with T7 RNA polymerase (Roche) and labeled with DIG. The primers are listed in Table S1. Linker primers were used to link the target fragment and T7, and SP6 promoter sequences; SP6‐forward primer 5′‐ATTAGGTGACACTATAGAAGGCCGCGG‐3′ and T7‐reverse primer 5′‐TAATACGACTCACTATAGGGCCCGGGGC‐3′. In situ hybridization analysis was performed following a previously published protocol (Hou et al. 2017). A total of 300 ng/mL DIG‐labeled RNA probes in hybridization buffer were applied and incubated at 55°C overnight. Signals were developed using NBT‐BCIP methods. Images were acquired using a Zeiss Axioscan 7 microscope slide scanner. Signals were measured using ImageJ software in a 100 µm‐width radial region in the medial and lateral areas. For E14.5 cortices, the regions covering the ventricular surface to the pia surface were selected. For E18.5 and P4 cortices, the cortical plate regions covering the pia surface to the subplate were selected. The intensity was relative to the mean value from four areas of the same ISH probe at the same time point.

### 2.12 Statistical Analysis

To compare whole brain GM volume between individuals with schizophrenia and healthy controls, we employed student's *t*‐tests. For voxel‐based comparison, the Generalized Linear Model (GLM) from the Statistics and Machine Learning Toolbox was utilized. The resulting *t* value of each GM voxel was constructed into a brain map named *t*‐map. Sex, age, and total intracranial volume were included as covariates in the GLM analyses. The GLM‐derived *t*‐maps were visualized using an xjView tool (https://www.alivelearn.net/xjview). The significant clusters of connected voxels were identified based on an uncorrected *p* value threshold of less than 0.001 at the single voxel level with a minimum voxel size of 100. A family‐wise error‐corrected *p* value of less than 0.05 at the cluster level was performed. In the permutation test, 60% of all participants were randomly selected for group comparisons 100 times. The top ten BAs were identified based on the rankings of the affected voxels (Figure 1). Pearson's correlation analysis was performed to investigate the relationships between clinical characteristics (e.g., duration of illness, MMSE, and PANSS) and the volumes of the whole brain, as well as the top 10 most affected BAs. In addition, two sample *t*‐tests were conducted to examine the differences in PRS.

## 3 Results

### 3.1 Decreased Whole Brain GM Volume in Individuals With Schizophrenia

In the healthy control group, the mean GM volume was 698.9417 cm^3^ (range: 562.76–778.65, SD: 32.8024), while that was 672.5034 cm^3^ (range: 536.53–748.12, SD: 36.79144). In individuals with schizophrenia (Figure S1), a significant difference was observed between the healthy control and individuals with schizophrenia (*p* = 2.59 × 10^−15^).

### 3.2 The Majority of the Disrupted Voxels Are Located in the BAs in the Ventral Frontal Cortex, Anterior Temporal Lobe, and Cingulate Cortex

**FIGURE 2:**
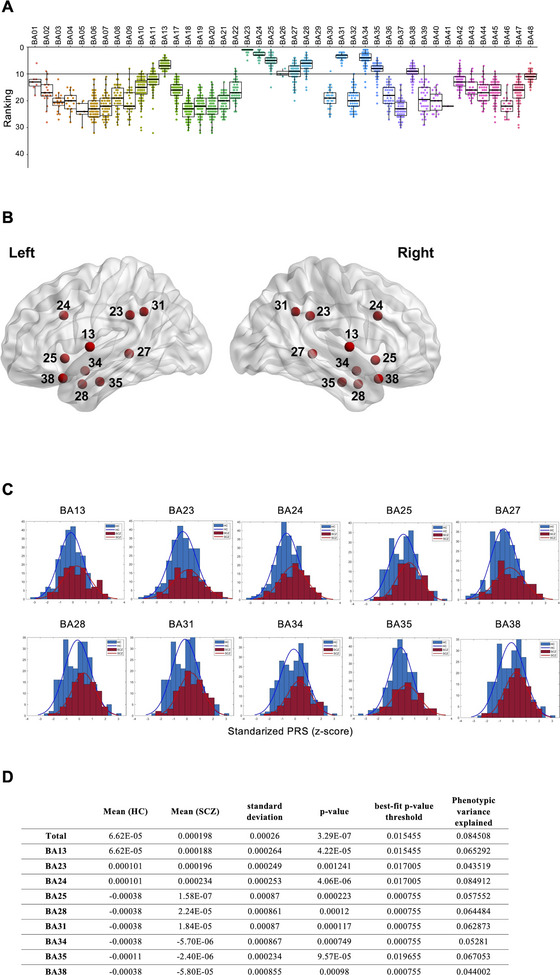
Functional areas with decreased gray matter volume in individuals with schizophrenia have a higher polygenic risk score. (A) The dot and box plots show the results of the permutation tests for gray matter volume change in the indicated Brodmann's areas. *N* = 100. (B) The render maps show the location of Brodmann's areas that exhibited the top ten reductions in gray matter volume. Numbers represent labels in Brodmann's area. (C) Bar plots show the distribution of standardized polygenic risk scores. The fitted normal distribution curves are shown as solid lines. Blue bars and lines represent the polygenic risk score results from healthy controls, and red bars and lines represent the polygenic risk score results from individuals with schizophrenia. (D) The table shows risk scores in healthy controls and individuals with schizophrenia, and the best‐fit *p*‐value threshold and phenotypic variance explained in each Brodmann's area. BA, Brodmann's area; HC, healthy controls; PRS, polygenic risk score; SCZ, schizophrenia.

The results showed that the affected voxels were widely distributed throughout the cerebrum, with notably higher intensity in the frontal‐temporal region and cingulate cortex (Figure 2A,B). We randomly selected 60% of the participants in each permutation test and conducted GLM analysis. After performing 100 rounds of permutation tests, we identified the top ten BAs based on the rank of affected voxels. The permutation analysis revealed that BA13, BA23, BA24, BA25, BA27, BA28, BA31, BA34, BA35, and BA38 consistently showed a higher proportion of affected voxels (Figure 2A,B). Thus, these BAs were selected as GWAS phenotypes for further investigation.

### 3.3 The Correlation Between Clinical Characteristics and Affected BA Areas in Individuals With Schizophrenia

The brain volume of the whole brain and the top ten affected BAs of individuals with schizophrenia showed a negative correlation with the duration of illness (*p* < 0.05 with a small correlation). We found a positive correlation between MMSE scores and GM volume in the whole brain (*p* = 0.004, *r* = 0.20), BA23 (*p* = 0.04, *r* = 0.15), BA24 (*p* = 0.02, *r* = 0.17), and BA34 regions (*p* = 0.004, *r* = 0.21). Notably, there was no significant correlation between GM volume and PANSS scores (Table S2).

### 3.4 Genetic Variants and the Mapped Genes Were Identified in the BAs With Changed GM Volume

**FIGURE 3:**
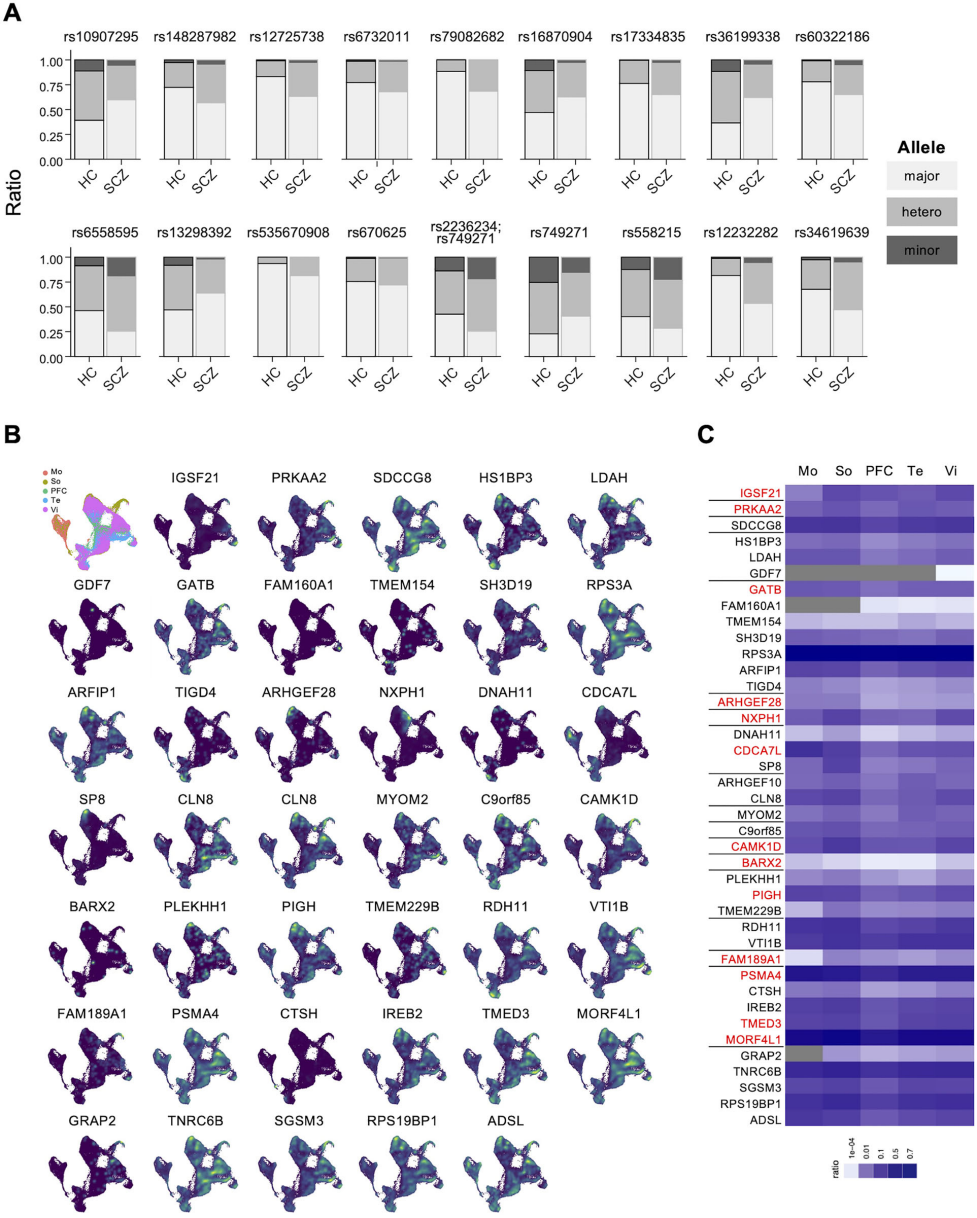
The spatial expression patterns of the schizophrenia‐associated genes in GW20 human neocortical cells. (A) Bar plots show the distribution of alleles of the indicated genetic variants in healthy controls and individuals with schizophrenia. HC, healthy controls; SCZ, schizophrenia. (B) UMAP plots of GW20 human neocortical cells from future motor, somatosensory, prefrontal cortex, temporal, and visual regions (Bhaduri et al. 2021); Plots are color‐coded according to cell source and the expression of indicated genes. (C) The heatmap shows the ratio of cells positive for the indicated genes in the indicated areas.

To screen for the possible genetic factors that might account for the decrease of GM volume in the identified BAs, we performed a genome‐wide association test, and the results were shown as a Manhattan plot (Figure S2). First, we tested the polygenic theory of schizophrenia in our dataset by calculating the polygenic risk score using PRSice. The individuals with schizophrenia with a significant decrease of GM volume in the identified BA were selected using a one‐sample *t*‐test with all healthy controls as the controls (Figure 2C). A two‐sample *t*‐test assessed the differences in PRS between cases and controls for each BA. The results showed that, in general, the schizophrenia group has a higher score compared to the healthy control group, and, except for BA38, the *p* values were smaller than the best‐fit *p* value threshold, suggesting that the BA‐associated SNPs have greater relevance in causing GM volume alterations in the identified BA of schizophrenia. In addition, the average phenotypic variance explained was 0.0627005 (range: 0.043519–0.084912, SD: 0.014297) (Figure 2D), explaining over 4% of the phenotypic variance in the schizophrenia group in each BA. Next, we seek the SNPs that have a higher potential to induce schizophrenia. We set a *p‐*value threshold (less than 5 × 10^−6^) to capture the potential SNPs. The results showed multiple genetic variants were changed in the individuals with schizophrenia (Figure 3A and Figure S2). The distribution of genetic variants, major and minor alleles, among healthy controls and individuals with schizophrenia differs (Figure 3A,B). In addition, we found that these genetic variants may share in multiple BAs or be unique to a certain BA. For example, we found rs12232282 on chromosome 15 and rs79082682 on chromosome 4 in almost all BAs except BA34, while only rs17334835 on chromosome 7 was found in BA34 (Table S3). This suggested that there might be a highly conserved combination of genetic factors responsible for schizophrenia in most cases. In contrast, a certain genetic cause may possibly be responsible for a certain type of schizophrenia. Next, we investigated the possible genes associated with the identified genetic variants via functional annotation and gene mapping using the FUMA GWAS server (https://fuma.ctglab.nl). Through positional mapping and eQTL mapping, we successfully identified 42 genes associated with identified variants (Table 1). In addition, as rs670625 is located on a predicted binding site for a transcription factor named *BARX2*, as reported in the JASPAR database (https://jaspar.genereg.net/) (Castro‐Mondragon et al. 2022), we included *BARX2* in our putative schizophrenia gene list.

**TABLE 1:** The SNP‐mapped genes.

rsID	CHR	BP	Odds ratio	*p* value	Major allele	Minor allele	Mapped gene
rs10907295	1	18536355	0.530	1.21E‐04	A	G	*IGSF21*
rs148287982	1	57082348	1.795	1.45E‐03	CAATA	C	*PPAP2B, PRKAA2*
rs12725738	1	242934793	2.625	9.72E‐06	G	A	*SDCCAG8*
rs6732011	2	21146521	1.527	3.98E‐02	T	G	*C2orf43, GDF7*
rs79082682	4	152285418	3.475	2.09E‐06	T	G	*PET112, FAM160A1*
rs16870904	5	73113650	0.547	3.56E‐04	T	C	*ARHGEF28*
rs17334835	7	8785903	1.782	4.76E‐03	C	T	*NXPH1*
rs36199338	7	21969746	0.437	1.45E‐06	AC	A	*DNAH11, CDCA7L, SP8*
rs60322186	8	1761339	1.936	8.32E‐04	C	T	*ARHGEF10, CLN8*
rs6558595	8	2028110	2.068	4.34E‐06	T	C	*MYOM2*
rs13298392	9	74611111	0.530	3.26E‐04	T	C	*C9orf85*
rs535670908	10	12605701	3.307	3.05E‐04	A	G	*CAMK1D*
rs670625	10	113635280	2.987	1.66E‐08	G	C	*BARX2*
rs2236234; rs749271	14	68038697	1.699	3.01E‐04	A	G	*PLEKHH1, PIGH, TMEM229B*
rs749271	14	68053149	0.581	2.10E‐04	G	A	*RDH11, VTI1B*
rs558215	15	29564635	1.587	1.65E‐03	T	C	*FAM189A1, NDNL2, GOLGA8T*
rs12232282	15	79136525	3.289	7.83E‐09	C	CA	*PSMA4, CTSH, IREB2, TMED3, MORF4L1*
rs34619639	22	40663874	2.092	4.85E‐05	A	C	*GRAP2, TNRC6B, SGSM3, RPS19BP1, ADSL*

Abbreviations: BP, base‐pair position; CHR, chromosome.

### 3.5 The Identified Schizophrenia‐Associated Genes Have Preferential Expression Patterns Among the Areas During Cortical Development

If the changes in the identified schizophrenia‐associated genes play roles in establishing schizophrenia‐associated BAs, these genes should theoretically have differential expression patterns among areas. To this, we performed in silico single‐cell RNA sequencing analysis of developing human neocortical cells from GW20 embryos when massive corticogenesis occurs and areal information appears (Bhaduri et al. 2021). Figure 3B displays UMAP‐annotated original cell sources across different brain regions, such as the motor, somatosensory, prefrontal, temporal, and visual cortex, providing an overview of the distribution of cells among areas. The expression profiles of the identified genes are depicted in Figure 3C. Multiple genes showed area‐preferential expression patterns, such as the higher expression of *MORF4L1* in the cells isolated from the future prefrontal and temporal lobes. Additionally, we included genes reported to be associated with schizophrenia. Based on these considerations, the following genes were selected for ISH analysis in mouse developing neocortex at multiple time points: *IGSF21, SDCCAG8, GATB, ARHGEF28, NXPH1, CDCA7L, CAMK1D, BARX2, PIGH, TMEM229B, FAM189A1, PSMA4, TMED3*, and *MORF4L1*. Among them, *SDCCAG8, GATB, CAMK1D, BARX2, PSMA4, TMED3*, and *MORF4L1* have higher odds ratios (> 2), higher associations with schizophrenia (*p *< 5 × 10^−6^) and are related to schizophrenia (Table 1) (GWAS catalog, https://www.ebi.ac.uk/gwas/home) (Kushima et al. 2018). *ARHGEF28*, *TMEM229B*, and *NXPH1* are associated with schizophrenia (GWAS catalog, https://www.ebi.ac.uk/gwas/home). *IGSF21, CDCA7L*, *PIGH*, and *FAM189A1* were selected due to their areal preferential expression patterns (Figure 3B,C).

### 3.6 The Dynamic Expression Pattern of the Selected Genes During Cortical Development

**FIGURE 4:**
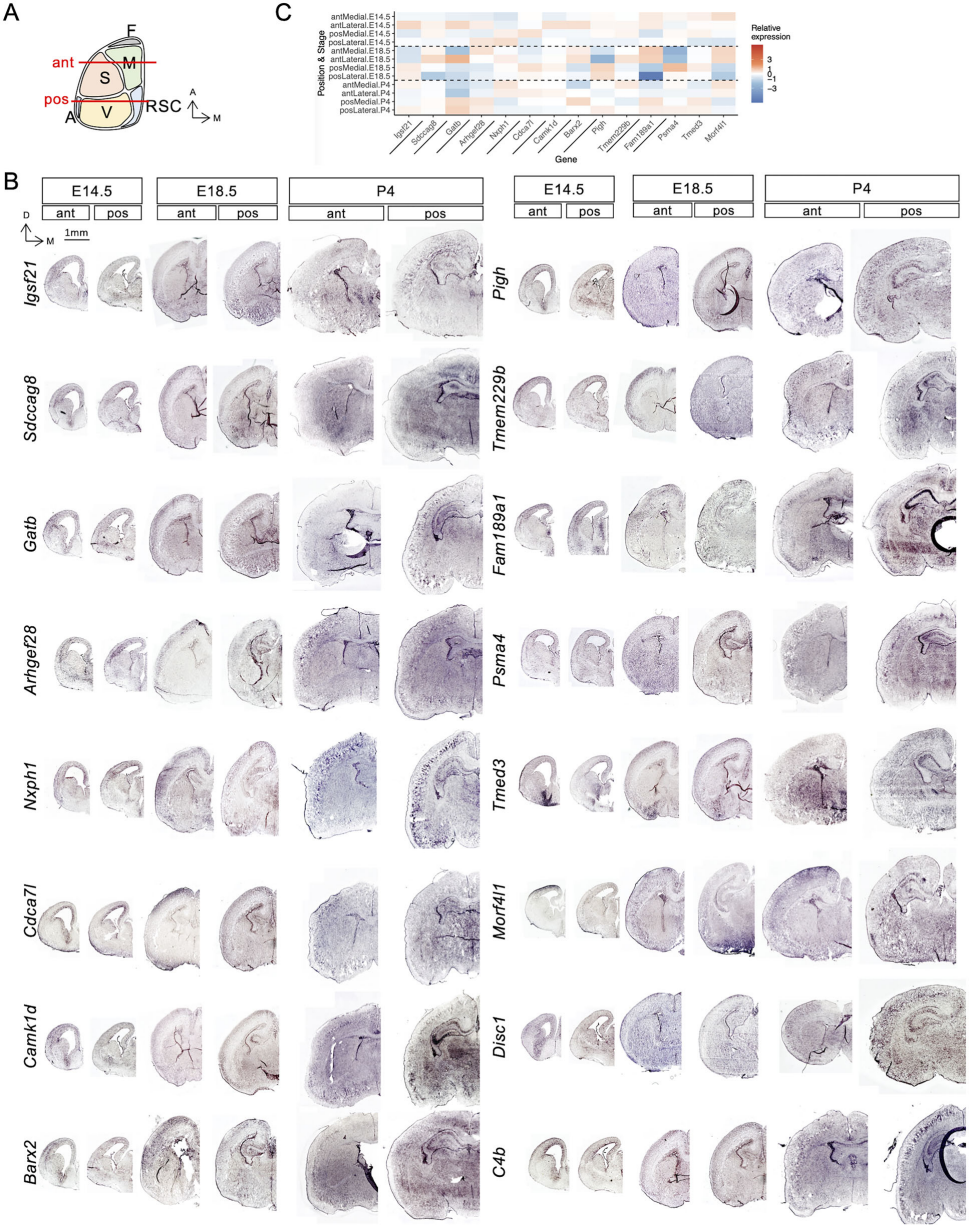
The temporal and spatial expression patterns of the schizophrenia‐associated genes in the developing mouse neocortex. (A) A schematic diagram shows the distribution of functional areas in the top view of the mouse neocortex. The positions of the anterior and posterior coronal planes are indicated. (B) In situ hybridization of the indicated genes in the developing mouse neocortex collected at E14.5, E18.5, and P4. Scale bars are indicated. (C) The heatmap shows the expression pattern of the indicated genes in the indicated positions of E14.5, E18.5, and P4 cortices. The signal intensity is related to the mean of signals from 4 regions at the same sample collection time. ant, anterior; pos, posterior.

We used in situ hybridization to investigate the spatial gene expression pattern of selected genes in the developing cortex of mice at E14.5, E18.5, and P4, representing the early, mid, and late stages of corticogenesis (Figure 4). Two coronal planes, anterior and posterior, were analyzed to cover future motor (medial) and somatosensory (lateral) areas in the anterior coronal sections, as well as future retrosplenial (medial) and visual (lateral) areas in the posterior coronal sections. We also included two reported schizophrenia‐related genes, *Disc1* (Thomson et al. 2016) and *C4b* (Sekar et al. 2016), to evaluate the effects of cortical arealization on schizophrenia. The results showed variable dynamic expression patterns across the genes, stage, and position (Figure 4B,C).

At E14.5, nine patterns were detected: *Igsf21, Camk1d, Barx2*, and *Gatb* in an anterolateral‐high pattern; *Psma4* in an anterolateral‐low pattern; *Disc1* in an anteromedial‐high pattern; *Tmed3* and *Pigh* in a posterolateral‐low pattern; *Morf4l1* and *Fam189al* in an anterior‐high posterior‐low pattern; *Nxph1* in a posterior‐high anterior‐low pattern; *Arhgef28* and *C4b* in a lateral‐high medial‐low pattern; *Cdca7l* in a medial‐high lateral‐low pattern; and *Sdccag8* and *Tmem229b* in an anterolateral and posteromedial‐high pattern. At E18.5, six patterns were detected: *Igsf21, Psma4, Pigh*, and *Cdca7l* in a posterior‐high anterior‐low pattern; *Morf4l1, Sdccag8, Arhgef28, Fam189a1, Barx2*, and *Disc1* in an anterior‐high posterior‐low pattern; *Tmed3* in a posteromedial‐high pattern; *Tmem229b* and *Gatb* in an anteromedial‐high pattern; *Nxph* in an anterolateral and posteromedial‐high pattern; and *C4b* in a medial‐high lateral‐low pattern. At P4, four patterns were detected: *Igsf21, Psma4, Pigh*, and *Tmem229b* in an anteromedial and posterolateral‐high pattern; *Tmed3, Sdccag8, Arhgef28, Fam189a1, Barx2, Gatb, Disc1*, and *C4b* in a posterior‐high anterior‐low pattern; *Morf4l1, Nxph1*, and *Cdca7l* in an anterior‐high posterior‐low pattern, and *Camk1d* in an anterolateral and posteromedial‐high pattern. Notably, genes mapped from the same SNP do not have similar expression patterns across all examined time points but may show similar or opposite expression patterns. For example, the rs12232282‐mapped genes *Psma4, Tmed3*, and *Morf4l1* showed opposite patterns, and the rs2236234;rs749271‐mapped genes *Pigh* and *Teme229b* showed the same pattern in P4 cortices (Figure 4C). Genes mapped from different SNPs may have similar expression patterns, such as *Sdccag8, Arhgef28, Fam189a1*, and *Disc1*, which showed similar patterns in E18.5 and P4 cortices.

## 4 Discussion

In this study, we detected several BAs with decreased GM volume in individuals with schizophrenia, in addition to the previous finding showing the global reduction of GM volume in individuals with schizophrenia (Gur et al. 1999). The identified BAs are located in the cortical lobes found in the previous findings (Bora et al. 2011; Shenton et al. 2001; Vita et al. 2012). These suggested that despite the heterogeneity of brain structural change in schizophrenia, some structural abnormalities in certain BAs may be particularly pathogenic for schizophrenic symptoms, such as hallucinations and delusions. According to the previous study, the sensory cortex, insula, putamen, and hippocampus may generate the neural circuit regarding hallucinations (Clos et al. 2014; Sommer et al. 2012), and the medial frontal cortex, anterior cingulate cortex, and insula may generate the neural circuit regarding delusions (Vicens et al. 2016). In our results, we detected decreased GM volume in BA13 in the insula, BA23, BA24, and BA31 in the cingulate cortex, and BA25 in the ventromedial frontal cortex. In addition, the differences in PRS showed that the identified BAs have significantly higher risk scores for schizophrenia compared to the control, and multiple genetic variants were observed. Interestingly, we found the same SNPs in these BAs, such as rs12232282 and rs79082682 in BA13, BA23, BA24, and BA31; rs670652 in BA13 and BA23; and rs6732011 in BA24 and BA31. Further, the dynamic expression profiles of the SNP‐mapped genes, such as *Psma4, Morf4l1, Gatb*, and *Barx2*, were detected. These results support the assumption that multiple genetic factors may regulate certain functional BAs, the deficiency of which could further possess heightened pathogenicity when it comes to schizophrenia.

The high complexity of connectomes within the human cerebral cortex establishes the complex behaviors in humans (Cohen et al. 2008), such as voluntary movement, decision‐making, social cognition, emotions, and attentional control, which, on the other hand, make it difficult to understand the pathological machinery underlying mental illness. Multiple areas are found to exert unique functions and are affected in individuals with schizophrenia. For example, the prefrontal cortex, crucial for executive functions, working memory, and decision‐making, often exhibits reduced GM volume abnormalities in schizophrenia (Kawada et al. 2009; Rusch et al. 2007; Selemon and Zecevic 2015). In addition to the previous finding showing that the ventromedial prefrontal cortex was affected in individuals with schizophrenia (Fan et al. 2013), we further identified the reduced GM volume in BA25, a subdivision of the ventromedial prefrontal cortex. As BA25 has strong connectivity with the amygdala and its functions in modulating negative emotion (Alexander et al. 2019), the deficiency of BA25 may explain the positive schizophrenia symptoms. The parahippocampal cortex is involved in visuospatial processing and episodic memory in the contextual processing of cognitive functions (Aminoff et al. 2013), and the subdivision, BA28 entorhinal cortex, is involved in memory, navigation, and the perception of time (Tsao et al. 2018). The smaller parahippocampal cortex and entorhinal cortex were observed in postmortem and MRI studies (Sim et al. 2006; Talbot and Arnold 2002). Our results indicated that, except for BA36, the entorhinal cortex, the GM volume in most subdivisions of the parahippocampal cortex, such as BA27, BA28, BA34, and BA35, was reduced, which may explain the impairment of episodic memory in schizophrenia (Guo et al. 2019; Zola‐Morgan et al. 1989). In line with previous findings (Herlin et al. 2021; Wright et al. 1999), we also detected a decrease in GM volume in the BA38 temporal pole in the temporal lobe of schizophrenia. The BA38 temporal pole serves as a hub for information confluence for visual cognition, social‐emotional processing, and semantic processing, and the deficiency induces auditory hallucination (Cosentino et al. 2010). The anterior cingulate cortex is involved in autonomic endocrine functions, emotion, and cognitive processing. In addition to previous reports showing the GM reduction in schizophrenia (Fornito et al. 2009), our results further identified the GM reduction in BA24, a subdivision of the anterior cingulate cortex, which particularly functions in emotion and cognition processing (Devinsky et al. 1995).

The pathogenesis of schizophrenia remains unclear, which may be due to the variable phenotypes and the polygenic causes. In our study, we found that the GM volume in several BAs was affected, and the associated SNPs and the SNP‐mapped genes were identified. We found that the dynamic expression pattern of some genes during cortical development matched the affected BAs found in the MRI analysis. For example, rs670625 is associated with BA13, BA23, and BA25, located in the middle lateral cortical regions and anteromedial and posteromedial cortical regions (Brodmann 1909). This SNP is on a predicted binding site of *BRAX2*, which might influence its ability to regulate downstream gene expression. In GW20 human cortical cells, *BARX2* is enriched in the future motor, somatosensory, and visual cortex, representing the relative anteromedial, anterolateral, and posterior cortical areas. In mice developing neocortex, *Barx2* is enriched in the anterolateral E14.5 cortex, the anteromedial and anterolateral E18.5 cortex, and the posterior P4 cortex. The similar expression pattern to the affected BAs implies the possible functions of Barx2 in schizophrenia. *BARX2* is a member of the homeobox transcription factor family expressed in the mouse‐developing CNS, such as the telencephalon, diencephalon, and mesencephalon (Jones et al. 1997). It can directly bind to the *NCAM* promoter to activate the expression and interact with CREB1 (Edelman et al. 2000), which is known to play roles in cortical development (Landeira et al. 2018). Combined with the dynamic expression profile, these data suggested that *BARX2/Barx2* is possible to regulate cortical development in the distinct areas that may be related to schizophrenia. Another example is CAMK1D, which is associated with the BA31 dorsal posterior cingulate area and is mapped from rs535670908. In mice, *Camk1d* is enriched in the posteromedial region of E18.5 cortices, while in the anterolateral region of E14.5 and P4 cortices.

The association test revealed 18 genetic loci associated with schizophrenia, and functional annotation identified 40 genes. Some of these genes, including *MORF4L1*, *PSMA4*, *ARHGEF28*, *TMEM229B*, *NXPH1*, *CAMK1D*, and *SDCCAG8*, have been reported to be associated with schizophrenia in previous studies (GWAS catalog, https://www.ebi.ac.uk/gwas/home/). In previous studies, the lack of Morf4l1 caused decreased progenitors and postmitotic neurons in the developing neural tube, resulting in a thinner neural tube in the E10.5 mouse forebrain (Chen et al. 2011; 2009). *MORF4L1* is part of the NuA4 histone acetyltransferase complex and the Sin3 complex, involved in transcriptional activation of specific genes principally by acetylation of nucleosomal histones H4 and H2A, suggesting *MORF4L1* controls cortical development via epigenetic mechanisms. *SDCCAG8* is a schizophrenia‐associated gene. Cells lacking *SDCCAG8* reveal distinctive expression patterns of genes associated with neurodevelopmental processes, including the generation of neurons and the organization of synapses (Flynn et al. 2020). *SDCCAG8* also regulates the accumulation of pericentriolar material at the centrosome and influences neuronal polarization and migration in the developing mouse cortex (Insolera et al. 2014), potentially impacting schizophrenia.

In this study, some unanswered questions remain to be addressed. First, this study included data from only one database. Future studies should incorporate additional cohorts to validate our findings. Second, it remains uncertain whether the application of antipsychotic medications would affect brain structure in individuals with schizophrenia or not. Third, the extent to which the mouse cerebral cortex models the human cerebral cortical development, as some of the functional areas do not exist in the mouse cerebral cortex.

## 5 Conclusions

In this study, we developed a comprehensive pipeline aimed at identifying impaired brain functional areas in individuals with schizophrenia. Through the integration of MRI data and an exploration of genetic factors using GWAS, we investigated the potential involvement of genetic components in the development of affected functional areas. Additionally, we examined the spatial and temporal expression patterns of the identified genes using single‐cell RNA sequencing analysis of the developing human cortical cells and in situ hybridization analysis of the developing mouse neocortex. By employing this analytical pipeline, we have made significant strides in uncovering genes closely linked to the affected functional areas in individuals with schizophrenia and provided insight into their potential roles in functional area development. Our research presents an approach to understanding the intricacies of impaired brain functional areas in schizophrenia, offering insights into the biological underpinnings of this mental illness.

## Author Contributions

**Jun‐Ding Zhu**: data curation, formal analysis, validation, visualization, writing – review and editing. **Chih‐Yun Chung**: data curation, formal analysis, methodology. **Shu‐Fei Lin**: data curation, formal analysis, visualization, validation. **Shih‐Jen Tsai**: resources, supervision, writing – review and editing. **Albert C. Yang**: writing – review and editing, conceptualization, methodology, resources, project administration, supervision, funding acquisition. **Pei‐Shan Hou**: conceptualization, data curation, formal analysis, methodology, project administration, supervision, validation, funding acquisition, writing – original draft, writing – review and editing, visualization.

## Conflicts of Interest

The authors declare no conflicts of interest.

## Ethics Statement

This study was conducted in accordance with the Declaration of Helsinki and was approved by the Institutional Review Board of Taipei Veterans General Hospital and National Yang Ming Chiao Tung University. All participants provided written informed consent.

## Peer Review

The peer review history for this article is available at https://publons.com/publon/10.1002/brb3.70688

## Supporting information




**Supplementary Materials**: brb370688‐sup‐0001‐SuppMat.pdf

## Data Availability

The datasets used and analyzed during the current study are available from the corresponding author on reasonable request.
