# Supplementary material for: Mapping Genetic Associations With Functional Brain Area Alterations in Schizophrenia and Implications for Cortical Development
Source: Brain Behav. 2025 Jul 20;15(7):e70688. doi: 10.1002/brb3.70688 (PMC12277548; doi:10.1002/brb3.70688)
Supplement: Supplementary file 1 — Supplementary Materials: brb370688‐sup‐0001‐SuppMat.pdf [file BRB3-15-e70688-s001.pdf]

Supplementary Materials

Mapping Genetic Associations with Functional Brain Area Alterations in Schizophrenia and Implications for Cortical Development

Jun-Ding Zhu, Chih-Yun Chung, Shu-Fei Lin, Shih-Jen Tsai, Albert C. Yang, Pei-Shan Hou

Supplementary Methods

Figure S1

Figure S2

Table S1

Table S2

Table S3

Supplementary Methods

Image acquisition and preprocessing

The T1-weighted MR scans were acquired by a 3T MRI scanner (Siemens Magnetom Tim Trio, Erlangen, Germany) with the following parameter settings: echo time = 3.5 ms; matrix size = 256 × 256; slices = 192; slice thickness = 1 mm; and voxel size = 1.0 × 1.0 × 1.0 mm^3. The raw images were processed using Data Processing Assistant for Resting-State fMRI (DPARSF) in the DPABI toolbox in MATLAB R2020b (Mathworks, Natick, MA, USA) (Yan, Wang, Zuo, & Zang, 2016). The preprocessing steps were as follows: (1) skull stripping was performed to remove non-brain tissue; (2) the raw images were reoriented based on the anterior commissure-posterior commissure line; (3) segmentation was performed to obtain GM, white matter and cerebrospinal fluid probability maps; (4) the images were normalized into the MNI152 standard space; (5) all preprocessed images were resampled with spatial voxel of 1.0 × 1.0 × 1.0 mm^3 resolution using a fsl-anat tool in the FSL library (https://fsl.fmrib.ox.ac.uk/fsl/fslwiki/FSL) (Jenkinson, Beckmann, Behrens, Woolrich, & Smith, 2012) (Figure 1A).

Brodmann map

Since the Brodmann interactive area atlas in MRIcro software lacks two areas (https://people.cas.sc.edu/rorden/mricro/mricro.html) (Rorden & Brett, 2000) (i.e., BA13 insula and BA31 dorsal posterior cingulate cortex), we extracted the information form Harvard-Oxford cortical and subcortical structural atlases (RRID:SCR_001476) and automated anatomical labeling atlas (AAL) (Rolls, Huang, Lin, Feng, & Joliot, 2020) to generate the modified Brodmann map. For insular cortex BA13, voxels corresponding to insular were extracted from the Harvard-Oxford Atlas. For area 31, we extracted the middle cingulate cortex (BA37 and BA38), which included partial BA23, BA24, and BA31, from the AAL and then filled the ventral cingulate cortex BA23 and BA24 from Brodmann interactive area. Thus, the remaining region was designated BA31 dorsal posterior cingulate cortex (Figure 1A).

GWAS genotype sample collection and quality control

To explore the genetic factors associated with affected BAs in individuals with schizophrenia versus healthy controls, a genome-wide association study was utilized. Individual genotypes were collected from the TAMI database. Axiom Genome-Wide TWB 1.0 Array Plate (Affymetrix), which is designed for Taiwanese Han individuals based on the GRch37/hg19 chromosome build and contains a total of 653,291 tagged SNPs, was utilized. The original genotypic files (.cel) were converted to PLINK format using the Axiom Analysis Suite v5.1 (Affymetrix) for the following quality control for the genome-wide association test.

We conducted quality control for false discoveries in GWAS. PLINK (version 1.9, http://pngu.mgh.harvard.edu/purcell/plink) (Purcell et al., 2007) and PLINK2 (version 2.0, www.cog-genomics.org/plink/2.0/) (Chang et al., 2015) were applied to filter low-quality samples, SNPs, and controlled the confounding variable, such as population structure. The QC protocol included the following steps and parameter settings. Samples and SNPs with a missing genotype rate above 0.02 were excluded. For sample

QC, samples with heterozygosity rates exceeding or falling below 3 standard deviations were removed to identify potential contamination or inbreeding. Then, we performed a sex check to identify samples with inconsistent reported and predicted sex. After linkage disequilibrium (LD) pruning ($r2 < 0.2$, 200 SNP windows), we identified and removed genetically related samples using the PLINK build-in function and then controlled for population stratification by performing PCA to extract the top 20 principal components, the results of which would be visualized using dot plots. SNP QC was conducted by excluding SNPs with a minor allele frequency (MAF) less than 0.05 and testing Hardy-Weinberg equilibrium (HWE) with different thresholds for control and case samples (--hwe 0.000001 for controls and --hwe 0.0000000001 for cases). After quality control, a total of 404 samples, with 232 being controls and 172 being cases (183 males and 221 females) and the number of SNPs decreased to 590,776.

Haplotype phasing and genotype imputation

The imputation process included chunking, separating the target chromosomes into small chunks using Imp5chunker, and imputing missing and untyped genotypes with Impute5 chunk by chunk. The required genetic recombination map was obtained from SHAPEIT4 (Delaneau, Zagury, Robinson, Marchini, & Dermitzakis, 2019). After imputation, the chunks were ligated into a whole chromosome using BCFtools (Danecek et al., 2021; Li, 2011), and variants with an INFO score less than 0.8 were filtered. After imputation, QC was applied to filter low-quality imputed variants with the following settings: --geno 0.02; --maf 0.05; --hwe 0.000001. We utilized PCA analysis to account for population stratification, and the dot plots confirmed the absence of population stratification in our dataset. Consequently, we did not include principal components as covariates during the association test.

Polygenic risk score

Polygenic risk scores (PRS) aim to quantify the cumulative effects of multiple genes or loci, condensing the information of various genetic variations into estimating an individual's genetic predisposition to a phenotype. In this study, PRS was applied to evaluate the contribution of genetic variants in each brain area (BA) to the risk of schizophrenia. The base data for PRS calculation, including the GWAS summary statistics and weight coefficients for each SNP, were obtained from publicly available data provided by the Psychiatric Genomics Consortium (https://www.med.unc.edu/pgc) (Lam et al., 2019). This dataset contained a large East Asian participant sample (22778 schizophrenia cases and 35362 controls). The base data underwent several QC steps, including filtering variants with a minor allele frequency (MAF) above 0.05, filtering variants with an INFO score lower than 0.8, and removing duplicate, mismatch, and ambiguous SNPs. The target data for each BA dataset underwent SNP QC steps as mentioned in the "Haplotype phasing and genotype imputation" section. Next, duplicate and mismatched SNPs were excluded (Choi, Mak, & O'Reilly, 2020). Later, SNP IDs were converted to rsID via SNPnexus (https://www.snp-nexus.org/v4/) (Oscanoa et al., 2020) and ANNOVAR (https://annovar.openbioinformatics.org/en/latest/) (Wang, Li, & Hakonarson, 2010). PRS calculations for the BAs datasets were performed using PRSice-2 software (Version 2.3.5, https://choishingwan.github.io/PRSice/)

(Choi & O'Reilly, 2019). The PRS underwent a linkage disequilibrium clumping process (250-kb window with an r2 cutoff of 0.1), and the 1000 Genomes Project phase 3v5 East Asian panel (also underwent SNPs ID conversion) served as the linkage disequilibrium reference. PRS was calculated for several p-value thresholds, and the best-fit p-value threshold was determined through regression analysis. The BA-based PRS was standardized and visualized using a "histfit" function in the Statistics and Machine Learning Toolbox on MATLAB.

Sample collection and cryosection

Institute of Cancer Research (ICR) mice were used in this study. The detection of a vaginal plug marked as embryonic day 0.5, E0.5, and the postnatal day 0, P0, corresponding to the day of birth. The mice were housed at the National Yang Ming Chiao Tung University Laboratory Animal Center following the guidelines of the Institutional Animal Care and Use Committee (IACUC). Brains were collected from three stages (E14.5, E18.5, and P4). Before brain collection, the E18.5 embryos and P4 mice were perfused with PBS and 4% PFA. The collected brains were stored in 4% PFA at 4°C, and the brains were transferred to 30% sucrose at 4°C at least one day before cryosectioning. The day after, the brains were washed with PBS and embedded in cryomatrix (Thermo). Serial coronal sections were performed with a thickness of 12 μm for embryonic brains and 14 μm for postnatal brains. After sectioning, the brain slices were stored at -80°C.

References

Chang, C. C., Chow, C. C., Tellier, L. C., Vattikuti, S., Purcell, S. M., & Lee, J. J. (2015). Second-generation PLINK: rising to the challenge of larger and richer datasets. *Gigascience, 4*, 7. doi:10.1186/s13742-015-0047-8

Choi, S. W., Mak, T. S., & O'Reilly, P. F. (2020). Tutorial: a guide to performing polygenic risk score analyses. *Nat Protoc, 15*(9), 2759-2772. doi:10.1038/s41596-020-0353-1

Choi, S. W., & O'Reilly, P. F. (2019). PRSice-2: Polygenic Risk Score software for biobank-scale data. *Gigascience, 8*(7). doi:10.1093/gigascience/giz082

Danecek, P., Bonfield, J. K., Liddle, J., Marshall, J., Ohan, V., Pollard, M. O., . . . Li, H. (2021). Twelve years of SAMtools and BCFtools. *Gigascience, 10*(2). doi:10.1093/gigascience/giab008

Delaneau, O., Zagury, J. F., Robinson, M. R., Marchini, J. L., & Dermitzakis, E. T. (2019). Accurate, scalable and integrative haplotype estimation. *Nat Commun, 10*(1), 5436. doi:10.1038/s41467-019-13225-y

Jenkinson, M., Beckmann, C. F., Behrens, T. E., Woolrich, M. W., & Smith, S. M. (2012). Fsl. *Neuroimage, 62*(2), 782-790. doi:10.1016/j.neuroimage.2011.09.015

Lam, M., Chen, C. Y., Li, Z., Martin, A. R., Bryois, J., Ma, X., . . . Huang, H. (2019). Comparative genetic architectures of schizophrenia in East Asian and European populations. *Nat Genet, 51*(12), 1670-1678. doi:10.1038/s41588-019-0512-x

Li, H. (2011). A statistical framework for SNP calling, mutation discovery, association mapping and

population genetical parameter estimation from sequencing data. *Bioinformatics, 27*(21), 2987-2993. doi:10.1093/bioinformatics/btr509

Oscanoa, J., Sivapalan, L., Gadaleta, E., Dayem Ullah, A. Z., Lemoine, N. R., & Chelala, C. (2020). SNPnexus: a web server for functional annotation of human genome sequence variation (2020 update). *Nucleic Acids Res, 48*(W1), W185-W192. doi:10.1093/nar/gkaa420

Purcell, S., Neale, B., Todd-Brown, K., Thomas, L., Ferreira, M. A., Bender, D., . . . Sham, P. C. (2007). PLINK: a tool set for whole-genome association and population-based linkage analyses. *Am J Hum Genet, 81*(3), 559-575. doi:10.1086/519795

Rolls, E. T., Huang, C. C., Lin, C. P., Feng, J., & Joliot, M. (2020). Automated anatomical labelling atlas 3. *Neuroimage, 206*, 116189. doi:10.1016/j.neuroimage.2019.116189

Rorden, C., & Brett, M. (2000). Stereotaxic display of brain lesions. *Behav Neurol, 12*(4), 191-200. doi:10.1155/2000/421719

Wang, K., Li, M., & Hakonarson, H. (2010). ANNOVAR: functional annotation of genetic variants from high-throughput sequencing data. *Nucleic Acids Res, 38*(16), e164. doi:10.1093/nar/gkq603

Yan, C. G., Wang, X. D., Zuo, X. N., & Zang, Y. F. (2016). DPABI: Data Processing & Analysis for (Resting-State) Brain Imaging. *Neuroinformatics, 14*(3), 339-351. doi:10.1007/s12021-016-9299-4

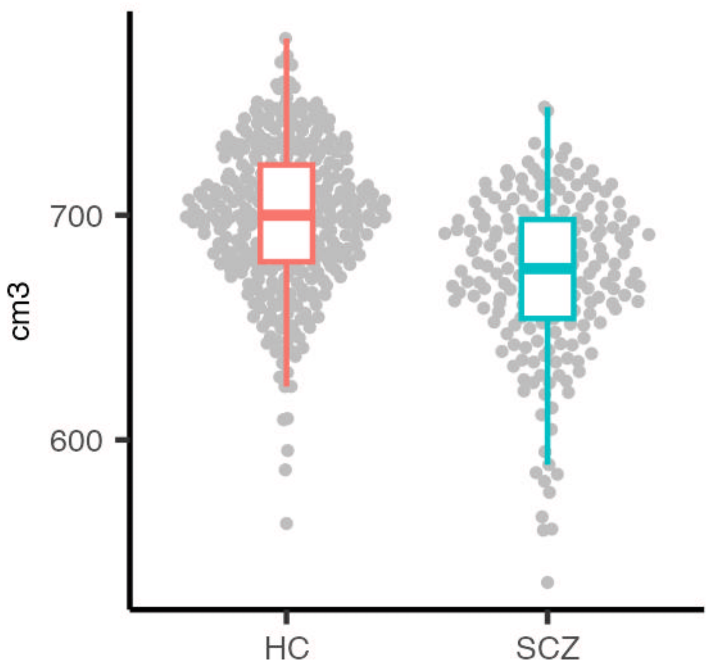

Figure S1. Smaller grey matter volume in patients with schizophrenia.
Box plot showing whole brain grey matter volume in healthy control (HC, left) and schizophrenia (SCZ, right). P value from student’s t-test is indicated.

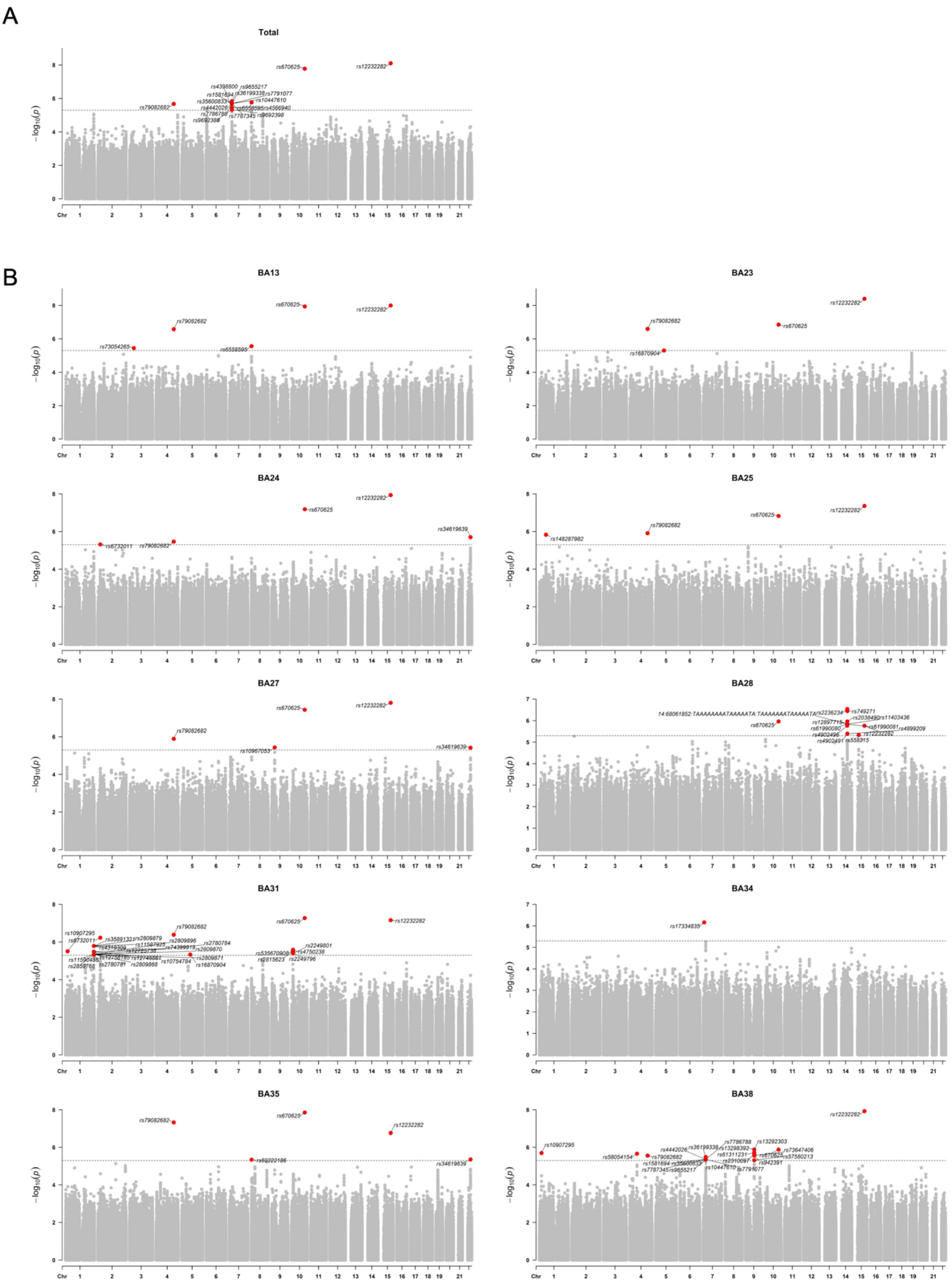

Figure S2. Schizophrenia-associated SNPs

(A) Manhattan plot showing SNPs association in GWAS analysis of all schizophrenia patients and all healthy controls. (B) Manhattan plots showing SNPs association in GWAS analysis of affected areas. Affected BAs are indicated.

Table S1. Primers for ISH probes.

Gene	Forward primer (5'→3')	Reverse primer (5'→3')
Cdca7l	GGCCGCGGATGCCGAGGATGAGTCCAGA	CCCGGGGCATGTCCACTTCGGATCCAGC
Arhgef28	GGCCGCGGCCAGGAGAAATCCCGCTACC	CCCGGGGCCTGTCTCTCCCTCTCCACCA
Tmem229b	GGCCGCGGCTCCTTGAGAAGACGGGACC	CCCGGGGCCCCTGTAATGCCCAAAGCGT
Nxph1	GGCCGCGGCATGGTTCCCTGTGAGAGAGC	CCCGGGGCAGAACAAGCTCCTCCCTCCAT
Morf4l1	GGCCGCGGCTTGTGTGCGTAGCGGTTA	CCCGGGGCTTCGGCTTAGGGTCCTGCTT
Pigh	GGCCGCGGGAGATGGACAAGGTCAAGGACAT	CCCGGGGCCTTGTTGCTGTGGCTTTCTGG
Fam189a1	GGCCGCGGTTGTCGCCTGTCCCACTAAC	CCCGGGGCGACTGTGACCTACCCAGCAC
Psma4	GGCCGCGGGTTTTGCTTGCAGCGGAGAG	CCCGGGGCTGCTCACAGGGAATTGGCTC
Igsf21	GGCCGCGGCCGTATGCGTGAGATCGTGT	CCCGGGGCGTGGCTCGGTCGTAGATACC
Gatb	GGCCGCGGCAGCTGAGAGTGGATGCCAA	CCCGGGGCCAGGTTGGGCTCTGGCATAA
Tmed3	GGCCGCGGACTTCCAAGTGGGTGACGAG	CCCGGGGCAGCCCTGTAGGAACCTGAGA
Barx2	GGCCGCGGCACCAGCCCCAGGAGTTATC	CCCGGGGCCGAGCACTTCCACCTACCTC
Disc1	GGCCGCGGTGCACTTTGCGGTTCATTCC	CCCGGGGCCGTGCCACATTCTGATTGCC
C4b	GGCCGCGGCTGAAGCCTCCAACGTTCCT	CCCGGGGCTGCCCACAGGTTGAAGGTTT
Sdccag8	GGCCGCGGAGGCTCCAGCAAGAACTGAAA	CCCGGGGCCAGAAGGTCAGTCTTCGCCT
Camk1d	GGCCGCGGTCCCAGAAGGGTTAAAGGGTAT	CCCGGGGCTAGATTCCTCCGATGTAAGGGA

Table S2. Correlation coefficients between cerebral grey matter volume of individuals with schizophrenia, and clinical characteristics.

Brain region	Duration of illness	MMSE-total	PANSS-P	PANSS-N	PANSS-total
**Whole brain**	-0.24*	0.20*	-0.05	-0.05	-0.03
**BA13**	-0.31*	0.08	-0.04	-0.03	-0.01
**BA23**	-0.23*	0.15*	-0.01	-0.05	-0.05
**BA24**	-0.22*	0.17*	-0.02	-0.09	-0.09
**BA25**	-0.21*	0.11	-0.03	0.003	0.002
**BA28**	-0.20*	0.14	-0.03	-0.04	-0.04
**BA31**	-0.26*	0.21*	0.10	-0.08	0.001
**BA34**	-0.29*	0.10	-0.07	0.04	0.001
**BA35**	-0.29*	0.14	-0.03	-0.02	-0.006
**BA38**	-0.24*	0.20*	-0.05	-0.05	-0.03

Abbreviation: MMSE, Mini-Mental Status Examination; PANSS, Positive and Negative Syndrome Scale; PANSS-P, PANSS positive scale; PANSS-N, PANSS negative scale; PANSS-total, PANSS total scale.

Significant correlations are indicated with * ($p < 0.05$).

Table S3. SNPs associated with the volume-decreased BAs in SCZ.

Total	BA13	BA23	BA24	BA25	BA27	BA28	BA31	BA34	BA35	BA38
rs6558595	rs6558595	rs6558595	rs12232282	rs12232282	rs12232282	rs12232282	rs12232282	rs17334835	rs12232282	rs12232282
rs12232282	rs12232282	rs12232282	rs79082682	rs79082682	rs79082682	rs2236234	rs12725738		rs79082682	rs79082682
rs79082682	rs79082682	rs79082682	rs34619639	rs148287982	rs34619639	rs749271	rs79082682		rs34619639	rs36199338
rs36199338	rs36199338	rs670625	rs6732011	rs670625		rs749271	rs10907295		rs60322186	rs10907295
	rs670625					rs558215	rs6732011			rs13298392
							rs535670908			
							rs16870904			

Abbreviation: BA, Brodmann areas; SCZ, schizophrenia.
